# Prenatal, Perinatal and Neonatal Risk Factors for Intellectual Disability: A Systemic Review and Meta-Analysis

**DOI:** 10.1371/journal.pone.0153655

**Published:** 2016-04-25

**Authors:** Jichong Huang, Tingting Zhu, Yi Qu, Dezhi Mu

**Affiliations:** 1 Department of Pediatrics, West China Second University Hospital, Sichuan University, Chengdu, 610041, China; 2 Key Laboratory of Obstetric & Gynecologic and Pediatric Diseases and Birth Defects of Ministry of Education, Sichuan University, Chengdu, 610041, China; 3 Department of Pediatrics, University of California San Francisco, San Francisco, CA, 94143, United States of America; Columbia University, UNITED STATES

## Abstract

**Background:**

The etiology of non-genetic intellectual disability (ID) is not fully known, and we aimed to identify the prenatal, perinatal and neonatal risk factors for ID.

**Method:**

PubMed and Embase databases were searched for studies that examined the association between pre-, peri- and neonatal factors and ID risk (keywords “intellectual disability” or “mental retardation” or “ID” or “MR” in combination with “prenatal” or “pregnancy” or “obstetric” or “perinatal” or “neonatal”. The last search was updated on September 15, 2015. Summary effect estimates (pooled odds ratios) were calculated for each risk factor using random effects models, with tests for heterogeneity and publication bias.

**Results:**

Seventeen studies with 55,344 patients and 5,723,749 control individuals were eligible for inclusion in our analysis, and 16 potential risk factors were analyzed. Ten prenatal factors (advanced maternal age, maternal black race, low maternal education, third or more parity, maternal alcohol use, maternal tobacco use, maternal diabetes, maternal hypertension, maternal epilepsy and maternal asthma), one perinatal factor (preterm birth) and two neonatal factors (male sex and low birth weight) were significantly associated with increased risk of ID.

**Conclusion:**

This systemic review and meta-analysis provides a comprehensive evidence-based assessment of the risk factors for ID. Future studies are encouraged to focus on perinatal and neonatal risk factors and the combined effects of multiple factors.

## Introduction

Intellectual disability (ID) or mental retardation (MR) is a developmental disability characterized by significant limitations in both intellectual functioning and adaptive behavior. Its onset occurs before 18 years of age[1], and its prevalence in the general population has been estimated at more than 1/100[2]. ID can be classified as genetic or non-genetic depending on its etiology. The causes of genetic ID, which accounts for only 30% to 50% of all ID cases[3], include chromosomal abnormalities (e.g. trisomy 21 syndrome), inherited genetic traits (e.g. fragile X syndrome) and single gene disorders (e.g. Prader—Willi syndrome)[4, 5]. However, the causes of non-genetic ID are not fully known. It is now suggested that the risk factors for non-genetic ID are extensive, and can be classified as prenatal, perinatal and neonatal factors according to the timing of suffering.

Numerous population-based studies have focused on specific non-genetic exposures as possible risk factors for ID. Many support the hypothesis that some prenatal (e.g. increasing maternal age, multiple gestation and maternal hypertension), perinatal (e.g. preterm birth and fetal distress) and neonatal (e.g. male sex, low birth weight and neonatal infection) exposures may increase the risk of ID[6, 7]. However, the overall conclusions of these studies are inconsistent. For example, some studies found that maternal hypertension increased the risk of ID[8, 9], whereas others did not[10–12]. The aim of the present study was to perform a meta-analysis of all pertinent available data to determine whether prenatal, perinatal, and neonatal exposures affect the onset of ID.

## Material and Methods

### Study identification and selection

The study was conducted according to the Preferred Reporting Items for Systematic Reviews and Meta-Analyses criteria (PRISMA) as shown in S1 File. The PubMed and Embase databases were searched using following keywords and subject terms: “intellectual disability”, “mental retardation”, “ID”, “MR” in combination with “prenatal”, “pregnancy”, “obstetric”, “perinatal”, “neonatal”. The last search was updated on September 15, 2015. There was no language restriction. All studies were initially screened by reading the titles and abstracts, and the full-texts of potential studies were independently assessed for eligibility by two reviewers (Jichong Huang and Tingting Zhu), who discussed disagreements to reach a consensus.

To be included in our analysis, the study had to: (1) evaluate the association between prenatal, perinatal, or neonatal factors and risk of ID, (2) have a cohort, case-control, or cross-sectional design, and (3) report odds ratios (ORs) and corresponding 95% confidence intervals (CIs) or other usable data. Studies were excluded if they: (1) did not eliminate the impact of genetic causes (e.g,. trisomy 21 syndrome, Prader-Willi syndrome and fragile X syndrome) on the risk of ID, (2) did not include a control group, or (3) overlapped with another study.

### Data extraction

The following data were independently collected by two reviewers (Jichong Huang and Tingting Zhu) from each of the included studies: name of the first author, year of publication, the country in which the study was conducted, study design, age at diagnosis, total number of cases and controls, exposure assessment methods, outcome assessment methods, ID diagnostic criteria, and risk factors.

We only included risk factors that were evaluated in two or more studies. For each risk factor, we extracted the OR together with the 95% CI. When the OR was not provided in the study, we computed a crude OR.

### Quality assessment

The methodological quality of each included study was independently assessed by two reviewers (Jichong Huang and Tingting Zhu) using the Newcastle-Ottawa Scale (NOS)[13, 14]. The quality score was evaluated based on three categories: group selection (four items), comparability between groups (one item), and outcome and exposure assessment (three items). And the maximum score was nine points. Studies were considered to be of high quality if they were scored above the median (> five points). Disagreements between the two reviewers were discussed until a consensus was reached.

### Statistical analysis

A separate meta-analysis was conducted for each risk factor and the pooled OR was calculated by using the fixed-effects model (a P-value >0.10 indicated heterogeneity) or the random-effects model (a P-value <0.10 indicated heterogeneity) [15, 16]. Heterogeneity among studies was assessed by using the chi-square and I^2^ tests. Data were considered statistically heterogeneous if I^2^ >50% and P was <0.1. Publication bias was assessed by conducting tests for funnel plot asymmetry[17]. Begg’s test[18] and Egger’s test[19] were also used to assess publication bias. The statistical tests were performed by using Review Manager 5.3 and STATA 12.0 software.

## Results

### Study selection and characteristics

**Fig 1:**
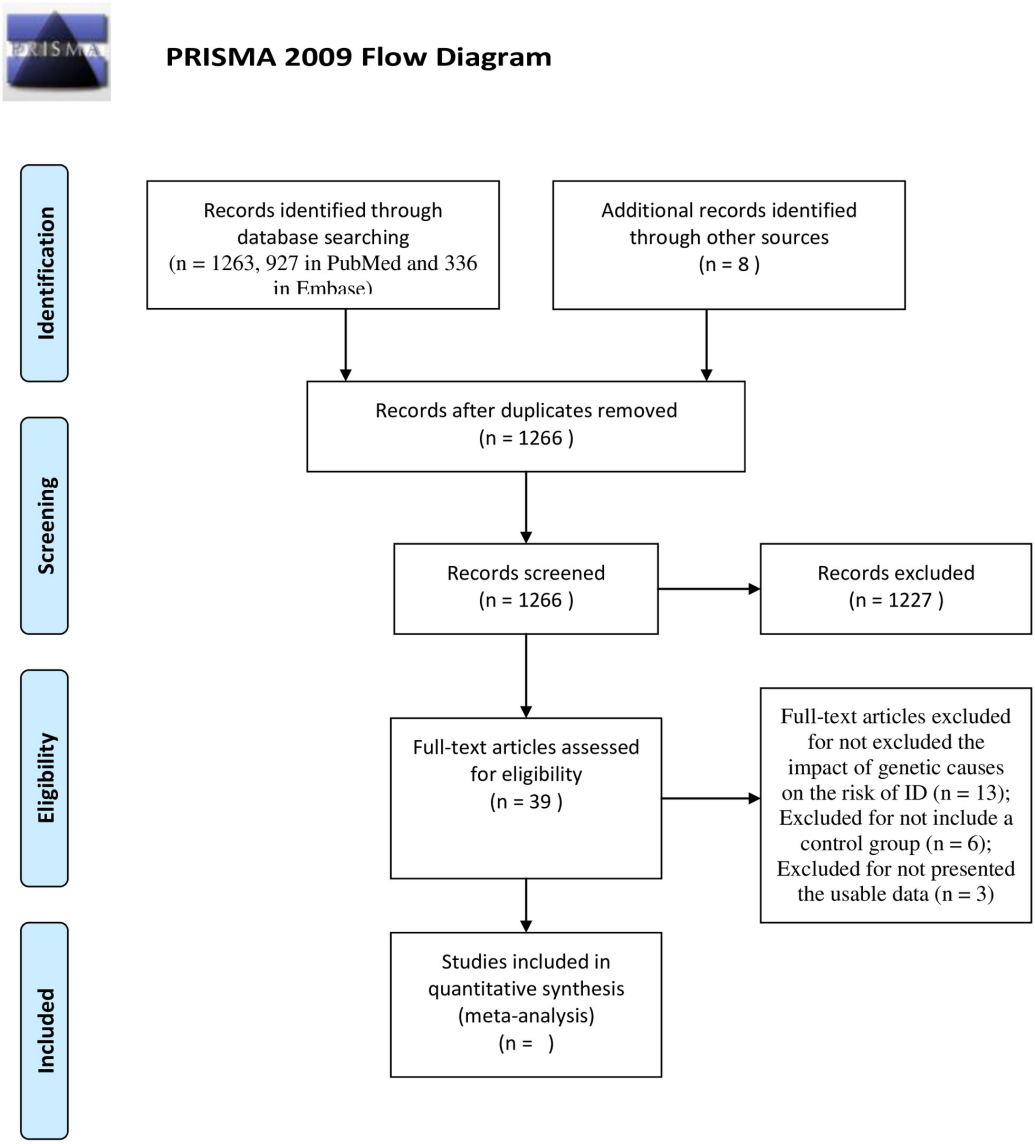
Flow gram of study selection process.

A total of 1,263 studies (927 in PubMed and 336 in Embase) and an additional eight studies culled from other sources were identified after an initial search. After reading the titles and abstracts, 1227 studies were excluded for irrelevant to the prenatal, perinatal or neonatal factors and ID risk, and five studies were excluded because of duplication. After reading the full-texts of the remaining 39 studies, 22 studies were excluded because they did not assess the effect of genetic causes on the risk of ID (13 studies), did not include a control group (six studies), or lacked usable data (three studies). Consequently, our analysis ultimately included 17 studies with a total of 55,344 patients and 5,723,749 control individuals. The meta-analysis included 15 risk factors (12 prenatal, one perinatal, and two neonatal). The selection process is shown in Fig 1, and the characteristics of each included study are presented in Table 1.

**Table 1:** Characteristics of the included studies in meta-analysis.

First author	Year	Country	Number(case/control)	Age of diagnosis	Study design	Exposure assessment	Outcome assessment	ID diagnostic criteria
Onicescu, G[20]	2014	USA	420/6859	6–11	Retrospective cohort study	Medical Record	Medical and school records	ICD9
Mann, JR[21]	2013	USA	3113/75562	3–6	Retrospective cohort study	Medical Record	Registration data	ICD9
Bilder, DA[11]	2013	USA	146/16936	8	Case-control study	Birth Certificate	School and health records	ICD9
O'Leary, C [22](Non-Aboriginal births)	2013	Australia	10576/30744	0–6	Retrospective cohort study	Medical record	Registration data	DSM-IV
O'Leary, C [22](Aboriginal births)	2013	Australia	7760/15762	0–6	Retrospective cohort study	Medical record	Registration data	DSM-IV
Langridge, AT[8]	2013	Australia	4576/376539	0–6	Retrospective cohort study	Medical Record; Birth Certificate	Registration data	DSM-IIIR/ IV/ IV-TR
Griffith, MI[9]	2011	USA	1636/79230	5–11	Retrospective cohort study	Medical Record; Birth Certificate	Registration data	ICD9
Mann, JR[10]	2012	USA	5780/159531	3–6	Retrospective cohort study	Medical Record; Birth Certificate	Registration data	ICD9
Mann, JR[23]	2009	USA	5388/129208	2–12	Retrospective cohort study	Medical Record; Birth Certificate	Registration data	ICD9
Leonard, H[12]	2006	Australia	2674/236964	7–16	Retrospective cohort study	Medical record	Registration data	DSM-IV
Croen, LA[24]	2001	USA	11114/4590333	0–12	Retrospective cohort study	Birth Certificate	Registration data	ICD9
Drews, CD[25]	1996	USA	221/400	10	Case-control study	Children's mother report	Registration data;School and health records	IQ≤70
Drews, CD[26]	1995	USA	316/563	10	Case-control study	Birth Certificate	Registration data;School and health records	IQ≤70
Decoufle, F[27]	1995	USA	314/562	10	Case-control study	Birth Certificate	Registration data;School and health records	IQ≤70
Yeargin-Allsopp, M[28]	1995	USA	330/563	10	Case-control study	Birth Certificate	Registration data;School and health records	IQ≤70
McDermott, S[29]	1993	USA	195/3098	5/9-10	Case-control study	Cildren's mother report	Registration data	Raven Progressive Matrices score≤70
Roeleveld, N[30]	1992	Netherlands	306/322	0–15	Case-control study	Medical Record	Medicaid Record;Registration data	ICD

Abbreviation:

ICD, International Classification of Diseases;

DSM: The Diagnostic and Statistical Manual of Mental Disorders;

IQ, Intelligence Quotient.

### Quality of the included studies

The methodological quality scores of the included studies ranged from four to seven points. The appraisal details are presented in S1 and S2 Tables. Ten of the 17 studies had scores of more than five points and were considered high quality. Variations in methodological quality may reflect in part the disproportionate inclusion of low-income families in some cohort studies[9, 10, 21, 23, 24] owing to the participation of these families in health insurance programs; consequently, middle- and upper-income families may have been underrepresented. In some case-control studies[25–29, 31], ID was only defined as intelligence quotient (IQ) ≤70, rather than via the diagnostic criteria of the International Classification of Diseases or the Diagnostic and Statistical Manual of Mental Disorders. Moreover, because ID can begin before the age of 18 years, the follow-up time was insufficient in most cohort studies.

### Prenatal, perinatal and neonatal risk factors

Tables 2–4 list the prenatal, perinatal, and neonatal risk factors included in the meta-analysis, as well as the summary effect estimate (ORs) with 95% CI and the P-value for each factor.

**Table 2:** Meta-analysis of prenatal risk factors for ID.

Risk factor	Authors	No. of case/total	OR (95% CI)	P-value*	P_Het_
	Bilder 2013	29/2086			
	Mann 2012	209/3583			
Advanced maternal age (≥35 vs.<35)	Mann 2009	51/2213	**1.53(1.35, 1.72)**	**<0.001**	0.03
	Croen 2001	1420/504751			
	Williams 1999	26/50			
	Drews 1996	32/112			
Advanced maternal age (≥30 vs.<30)	Drews 1995	NR	0.81(0.62, 1.05)	0.11	0.57
	Yeargin-Allsopp 1995	53/152			
	Bilder 2013	19/2266			
	Mann 2009	10/906			
Maternal age<20	Williams 1999	132/256	1.15(0.98, 1.35)	0.09	0.2
	Drews 1996	60/129			
	Drews 1995	NR			
	Yeargin-Allsopp 1995	86/204			
	Onicescu 2014	1443/38204			
	Mann 2013	2892/81230			
	Mann 2012	321/4873			
Maternal black race	Croen 2001	1650/367611	**1.08(1.04, 1.13)**	**<0.001**	<0.001
	Williams 1999	356/624			
	Drews 1996	170/351			
	Drews 1995	NR			
	Yeargin-Allsopp 1995	232/499			
	Mann 2013	2399/60184			
	Mann 2012	1312/29950			
	Griffith 2011	749/31988			
	Mann 2009	2247/47540			
Low maternal education (<12 vs.≥12 years)	Williams 1999	439/848	**1.33(1.28, 1.37)**	**<0.001**	<0.001
	Drews 1996	121/198			
	Drews 1995	NR			
	Decoufle 1995	176/332			
	Yeargin-Allsopp 1995	177/333			

*: Significant positive results (P<0.05).

NR, Not Report.

**Table 3:** Meta-analysis of prenatal risk factors for ID (Continued).

Risk factor	Authors	No. of case/total	OR (95% CI)	P-value*	P_Het_
	Croen 2001	3258/1412612			
Parity = 2 (2 vs.1)	Drews 1996	71/236	1.11(0.96, 1.28)	0.15	0.49
	Drews 1995	NR			
	Yeargin-Allsopp 1995	95/307			
	Croen 2001	4148/1347012			
Parity≥3 (≥3 vs.1)	Drews 1996	79/158	**1.63(1.45, 1.84)**	**<0.001**	0.22
	Drews 1995	NR			
	Yeargin-Allsopp 1995	119/238			
	Onicescu 2014	8/85			
	O'leary 2013 (Non-Aboriginal births)	265/676			
Maternal alcohol use	O'leary 2013 (Aboriginal births)	358/811	**1.63(1.49, 1.78)**	**<0.001**	0.02
	Mann 2009	68/1116			
	Drews 1996	39/104			
	Roeleveld 1992	204/383			
	Onicescu 2014	77/1180			
	Mann 2013	1177/30392			
	Bilder 2013	13/1389			
Maternal tobacco use	Mann 2012	629/14503	**1.10(1.06, 1.15)**	**<0.001**	0.53
	Mann 2009	1089/27169			
	Drews 1996	221/400			
	Roeleveld 1992	154/308			
	Langridge 2013	NR			
Maternal diabetes	Mann 2013	734/17988	**1.15(1.08, 1.23)**	**<0.001**	0.01
	Mann 2012	420/9784			
	Leonard 2006	47/2680			
	Bilder 2013	6/654			
	Langridge 2013	NR			
Maternal hypertension /pre-eclampsia/eclampsia	Griffith 2011	155/5169	**1.33(1.24, 1,42)**	**<0.001**	<0.001
	Mann 2012	523/11416			
	Leonard 2006	17/1379			
Maternal epilepsy	Mann 2012	29/333	**2.76(2.16, 3.52)**	**<0.001**	0.03
	Leonard 2006	42/1147			
Maternal asthma	Langridge 2013	NR	**1.48(1.24, 1.75)**	**<0.001**	0.75
	Leonard 2006	126/7743			

*: Significant positive results (P<0.05).

NR, Not Report.

**Table 4:** Meta-analysis of peri- and neonatal risk factors for ID.

Risk factor	Authors	No. of case/total	OR (95% CI)	P-value*	P_Het_
Preterm birth (GA<37 vs.≥37 weeks)	Bilder 2013	32/1359	**2.03(1.79, 2.31)**	**<0.001**	0.06
	Griffith 2011	NR			
	Onicescu 2014	284/3713			
	Langridge 2013	NR			
	Mann 2013	4028/83485			
	Mann 2012	2141/39669			
	Griffith 2011	1106/1174			
Male sex	Croen 2001	7027/2350287	**1.96(1.90, 2.01)**	**<0.001**	<0.001
	Mann 2009	3620/68610			
	Williams 1999	289/566			
	Drews 1996	138/333			
	Drews 1995	NR			
	Yeargin-Allsopp 1995	199/466			
	Bilder 2013	30/868			
	Griffith 2011	349/7667			
Low birth weight (<2500g vs.≥2500g)	Croen 2001	8537/4314859	**3.56(3.27, 3.89)**	**<0.001**	<0.001
	Drews 1996	39/73			
	McDermott 1993	195/3293			

*: Significant positive results (P<0.05).

NR, Not Report.

The statistically significant prenatal risk factors identified in the meta-analysis were as follows: advanced maternal age (maternal age over 35) (OR, 1.54; 95% CI, 1.21 to 1.96), maternal black race (OR, 1.71; 95% CI, 1.33 to 2.20), low maternal education (OR, 2.09; 95% CI, 1.73 to 2.52), third or more parity (OR, 1.63; 95% CI, 1.45 to 1.84), maternal alcohol use (OR, 1.54; 95% CI, 1.30 to 1. 83), maternal tobacco use (OR, 1.10; 95% CI, 1.06 to 1.15), maternal diabetes (OR, 1.19; 95% CI, 1.00 to 1.41), maternal hypertension (OR, 1.28; 95% CI, 1.01 to 1.64), maternal epilepsy (OR, 2.63; 95% CI, 1.54 to 4.49) and maternal asthma (OR, 1.48; 95% CI, 1.24 to 1.75) (Table 2). There was heterogeneity among the individual studies for most outcomes excepting those for third or more parity (I^2^ = 32%, P = 0.22), maternal tobacco use (I^2^ = 0%, P = 0.53), and maternal asthma (I^2^ = 0%, P = 0.75) (Table 2).

Significant positive associations were also found between ID risk and preterm birth (a prenatal factor) (OR, 2.50; 95% CI, 1.38 to 4.53) and between ID risk and two neonatal factors: male sex (OR, 1.84; 95% CI, 1.64 to 2.07) and low birth weight (OR, 3.43; 95% CI, 2.25 to 5.32) (Table 3). Individual studies were heterogeneous for both neonatal outcomes (I^2^ = 93%, P <0.0001 for both) (Table 3).

### Publication bias

**Fig 2:**
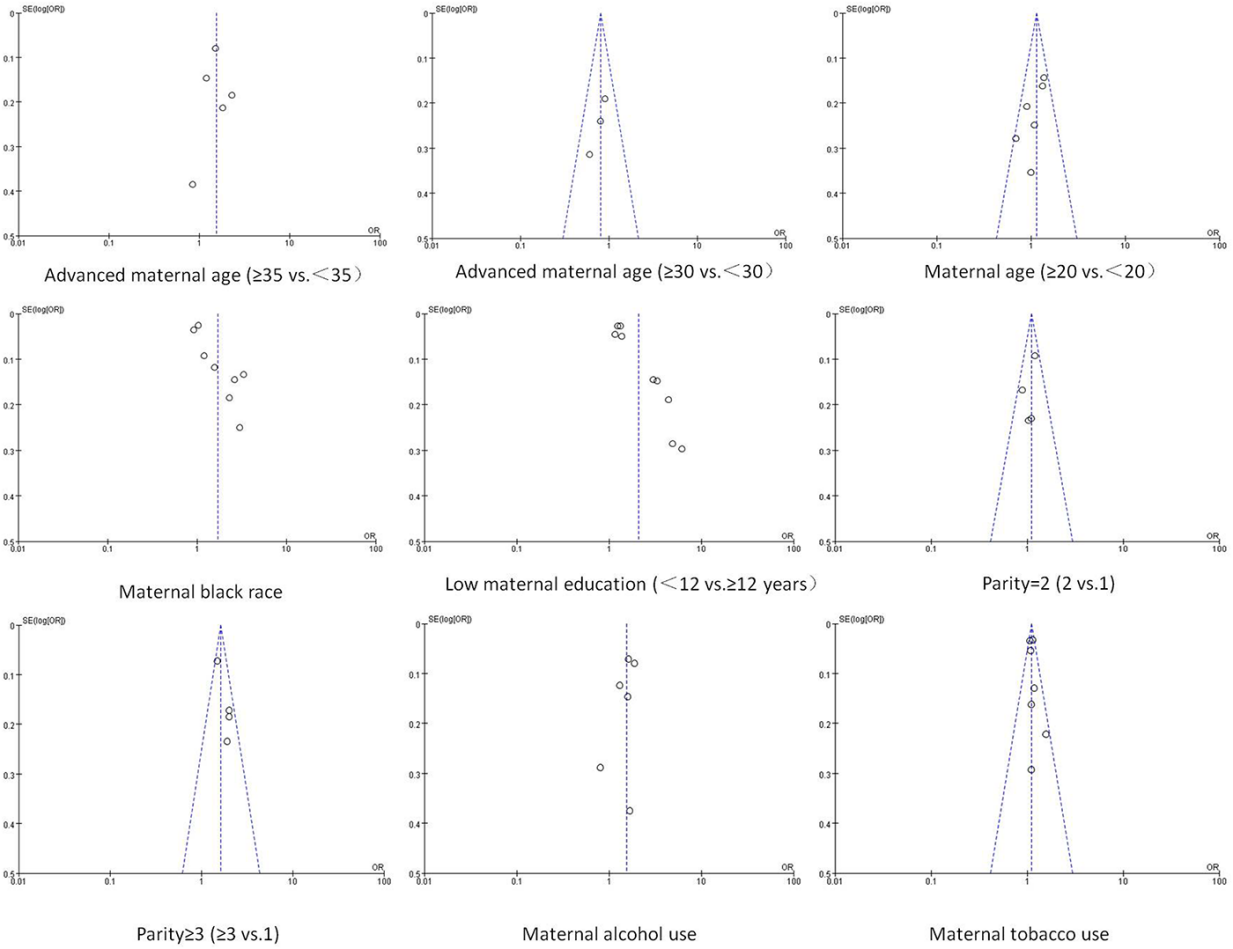
Funnel plot for publication bias test. The two oblique lines indicate the pseudo 95% confidence limits.

**Fig 3:**
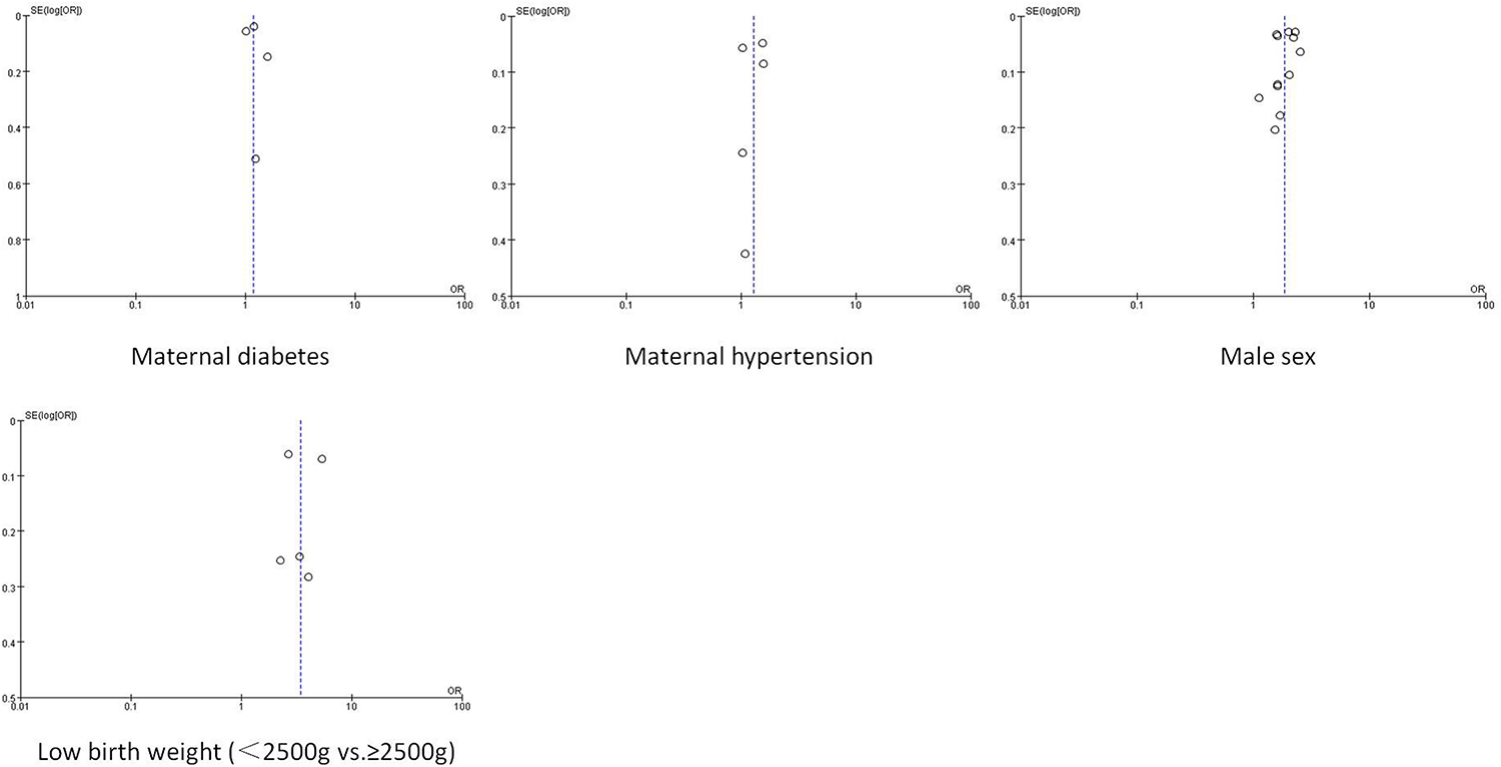
Funnel plot for publication bias test (Continued). The two oblique lines indicate the pseudo 95% confidence limits.

Publication bias was assessed for the factors examined in the pooled analysis of more than two studies (Figs 2 and 3). Significant publication bias was found for maternal black race (Begg’s test, P = 0.048; Egger’s test, P = 0.006) and low maternal education (Begg’s test, P = 0.022; Egger’s test, P <0.001).

## Discussion

Identifying patients with a high risk of non-genetic ID is important for early detection and intervention of ID, which would benefit both clinical practice and public health. To our knowledge, this study is the first systemic review and meta-analysis of the relationship between prenatal, perinatal, and neonatal factors and the risk of ID. This meta-analysis included 17 cohort or case-control studies with a total of 55,344 patients and 5,723,749 control individuals.

The factors strongly associated with ID risk were advanced maternal age (>35 years), maternal black race, low maternal education, third or more parity, maternal alcohol use, maternal epilepsy, preterm birth, male sex, and low birth weight. Other significant factors with a lower strength of association (risk estimates less than 1.5) were maternal tobacco use, maternal diabetes, maternal hypertension and maternal asthma.

The fetus is highly susceptible to changes in maternal conditions. Risk factors such as maternal toxicant exposure and metabolic disorders can significantly affect the genetically programmed brain development of the fetus. Previous studies have shown that maternal tobacco exposure[32], alcohol exposure[33], diabetes[34] and hypertension[35] can result in abnormal brain development. These risk factors are therefore closely associated with intelligence development, as indicated by our meta-analysis. What’s more, Mann et al. have demonstrated that pre-pregnancy obesity contributes to maternal metabolic disorders, and is also associated with significantly increased risk of ID in the children[21]. Therefore, more epidemiological studies in a variety of geographic groups are needed in the future to test the consistency and generalisability of this association.

Before the 21st century, advanced maternal age was considered to be >30 years. Several studies during that period investigated but could not confirm a relationship between maternal age and risk of ID owing to insignificant results[25, 26, 28]. In recent years, the maternal age at delivery has increased, and advanced maternal age has been redefined as >35 years. Our meta-analysis demonstrates a positive association between maternal age >35 years and the risk of ID. The father’s age at the time the of child’s birth is also increasing, and has been shown to correlate with ID risk[30]. However, most studies in our meta-analysis did not exclude the potential effects of this confounder. Further well-adjusted studies are needed and should explore the other cut-off criteria besides age over 35-year-old.

There were no significant associations between ID risk and young maternal age (under 20-year-old) or second parity. The reason may be the limited number of included studies and insufficient simple size. Young maternal age often indicates a low education level, which is also related to ID risk. However, some of the studies included in our meta-analysis did not control this confounder. Moreover, since young maternal age ranges from 16 to 20 years old[36, 37], a more extensive age range should be taken into consideration in future investigations of ID risk.

Two previous meta-analyses examined the relationship between IQ score and preterm birth[38, 39]. Both reported significantly lower IQ scores in preterm as compared with full-term infants, which is consistent with our results. In addition, Kerr-Wilson et al. reported a linear dose-response relationship between the IQ score and the gestation period: the IQ score decreased steadily for each 1-week decrease in the gestation period[38]. Similarly, it is worthwhile to determine whether this relationship exists even in ID cases with different severity levels. Therefore, further studies should be performed to investigate the associations between different gestational weeks and the severity of ID in preterm infants.

Some limitations of this meta-analysis should be addressed. First, several studies presented only crude ORs and did not adjust for other potential confounders, which might have resulted in overestimation of the association between risk factors and ID. Second, the heterogeneity among studies was noted in most risk factors. This heterogeneity resulted from the different diagnostic criteria of ID, age of diagnosis, sample size, and exposure or outcome assessment methods among studies. Moreover, the number of studies in each meta-analysis limited our ability to investigate these factors. Third, only published data were included in this analysis.

## Conclusions

This systemic review and meta-analysis identified prenatal, perinatal and neonatal risk factors for ID. The findings of this study indicate that prenatal factors have been the focus for studies on ID risk. In the future, more studies that focus on perinatal and neonatal factors are required, and researchers should be encouraged to investigate combinations of factors to a further study on the mechanisms leading to ID.

## Supporting Information

S1 File PRISMA checklist. (DOC)

S2 File Reasons for Exclusion. (DOCX)

S1 Table New Castle Ottawa (NOS) quality assessment for cohort studies. (XLSX)

S2 Table New Castle Ottawa (NOS) quality assessment for case-control studies. (XLSX)
