# Supplementary material for: Prenatal, Perinatal and Neonatal Risk Factors for Intellectual Disability: A Systemic Review and Meta-Analysis
Source: PLoS One. 2016 Apr 25;11(4):e0153655. doi: 10.1371/journal.pone.0153655 (PMC4844149; doi:10.1371/journal.pone.0153655)
Supplement: S2 File — (DOCX) [file pone.0153655.s002.docx]

# Reasons for Exclusion

**Exclusion for not excluded the impact of genetic causes on the risk of ID (13 references):**

1. Totsika V, Hastings RP, Emerson E, Lancaster GA, Berridge DM. A population-based investigation of behavioural and emotional problems and maternal mental health: associations with autism spectrum disorder and intellectual disability. Journal of child psychology and psychiatry, and allied disciplines. 2011;52(1):91-9.

2. Jauhari P, Boggula R, Bhave A, Bhargava R, Singh C, Kohli N, et al. Aetiology of intellectual disability in paediatric outpatients in Northern India. Dev Med Child Neurol. 2011;53(2):167-72.

3. 1966 cohort . Heikura U, Taanila A, Hartikainen AL, Olsen P, Linna SL, von Wendt L, et al. Variations in prenatal sociodemographic factors associated with intellectual disability: a study of the 20-year interval between two birth cohorts in northern Finland. American journal of epidemiology. 2008;167(2):169-77.

4. 1986 cohort . Heikura U, Taanila A, Hartikainen AL, Olsen P, Linna SL, von Wendt L, et al. Variations in prenatal sociodemographic factors associated with intellectual disability: a study of the 20-year interval between two birth cohorts in northern Finland. American journal of epidemiology. 2008;167(2):169-77.

5. Altunbasak S, Incecik F, Herguner O, Refik Burgut H. Prognosis of patients with seizures occurring in the first 2 years. Journal of child neurology. 2007;22(3):307-13.

6. Durkin MS, Khan NZ, Davidson LL, Huq S, Munir S, Rasul E, et al. Prenatal and postnatal risk factors for mental retardation among children in Bangladesh. American journal of epidemiology. 2000;152(11):1024-33.

7. Cans C, Wilhelm L, Baille MF, du Mazaubrun C, Grandjean H, Rumeau-Rouquette C. Aetiological findings and associated factors in children with severe mental retardation. Dev Med Child Neurol. 1999;41(4):233-9.

8. Durkin MS, Hasan ZM, Hasan KZ. Prevalence and correlates of mental retardation among children in Karachi, Pakistan. American journal of epidemiology. 1998;147(3):281-8.

9. Waugh J, O'Callaghan MJ, Tudehope DI, Mohay HA, Burns YR, Gray PH, et al. Prevalence and aetiology of neurological impairment in extremely low birthweight infants. Journal of paediatrics and child health. 1996;32(2):120-4.

10. Whitaker AH, Feldman JF, Van Rossem R, Schonfeld IS, Pinto-Martin JA, Torre C, et al. Neonatal cranial ultrasound abnormalities in low birth weight infants: relation to cognitive outcomes at six years of age. Pediatrics. 1996;98(4 Pt 1):719-29.

11. Jonas O, Roder D, Esterman A, Macharper T, Chan A. Pregnancy and birth risk factors for intellectual disability in South Australia. European journal of epidemiology. 1989;5(3):322-7.

12. Lamont MA, Dennis NR. Aetiology of mild mental retardation. Archives of disease in childhood. 1988;63(9):1032-8.

13. Rantakallio P, von Wendt L. Risk factors for mental retardation. Archives of disease in childhood. 1985;60(10):946-52.

**Exclusion for** **not include a control group (6 references):**

1. Cheung EN, George SR, Andrade DM, Chow EW, Silversides CK, Bassett AS. Neonatal hypocalcemia, neonatal seizures, and intellectual disability in 22q11.2 deletion syndrome. Genetics in medicine : official journal of the American College of Medical Genetics. 2014;16(1):40-4.
2. Tsai WH, Hwang YS, Hung TY, Weng SF, Lin SJ, Chang WT. Association between mechanical ventilation and neurodevelopmental disorders in a nationwide cohort of extremely low birth weight infants. Research in developmental disabilities. 2014;35(7):1544-50.
3. Heikura U, Linna SL, Olsen P, Hartikainen AL, Taanila A, Jarvelin MR. Etiological survey on intellectual disability in the northern Finland birth cohort 1986. American journal of mental retardation : AJMR. 2005;110(3):171-80.
4. Heikura U, Taanila A, Olsen P, Hartikainen AL, von Wendt L, Jarvelin MR. Temporal changes in incidence and prevalence of intellectual disability between two birth cohorts in Northern Finland. American journal of mental retardation : AJMR. 2003;108(1):19-31.
5. Hou JW, Wang TR, Chuang SM. An epidemiological and aetiological study of children with intellectual disability in Taiwan. Journal of intellectual disability research : JIDR. 1998;42 ( Pt 2):137-43.
6. Benassi G, Guarino M, Cammarata S, Cristoni P, Fantini MP, Ancona A, et al. An epidemiological study on severe mental retardation among schoolchildren in Bologna, Italy. Dev Med Child Neurol. 1990;32(10):895-901.

**Exclusion for** **not presented the usable data (3 references):**

1. Morsing E, Marsal K. Pre-eclampsia- an additional risk factor for cognitive impairment at school age after intrauterine growth restriction and very preterm birth. Early human development. 2014;90(2):99-101.

2. Ronen GM, Buckley D, Penney S, Streiner DL. Long-term prognosis in children with neonatal seizures: a population-based study. Neurology. 2007;69(19):1816-22.

3. Camp BW, Broman SH, Nichols PL, Leff M. Maternal and neonatal risk factors for mental retardation: defining the 'at-risk' child. Early human development. 1998;50(2):159-73.
